# Internet-delivered cognitive behaviour therapy for affective disorders, anxiety disorders and somatic conditions: An updated systematic umbrella review

**DOI:** 10.1177/20552076241287643

**Published:** 2024-10-07

**Authors:** Anton Käll, Ieva Biliunaite, Gerhard Andersson

**Affiliations:** 1 Department of Behavioural Sciences and Learning, 4566 Linköping University, Linköping, Sweden; 2 Department of Biomedical and Clinical Sciences, 4566 Linköping University, Linköping, Sweden; 3 4501 Leiden University, Leiden, The Netherlands; 4 Department of Clinical Neuroscience, 27106 Karolinska Institutet, Stockholm, Sweden

**Keywords:** ICBT, internet intervention, eHealth, cognitive behaviour therapy, psychiatric disorders, somatic disorders

## Abstract

**Background:**

Internet-delivered cognitive behaviour therapy (ICBT), which can involve guidance from a clinician, continues to be a way to deliver psychological treatments. A previous umbrella review identified moderate-to-large effect sizes favouring ICBT compared to control conditions when treating major depression and anxiety disorders. Given the rapid developments in the field, an updated umbrella review of available meta-analyses can show other conditions and subpopulations for which ICBT is effective. The aim of the study was to provide an expanded overview of the efficacy of ICBT for a broader range of adult psychiatric and somatic conditions.

**Methods:**

We conducted an updated search of the literature since the publication of the previous umbrella review back in 2019 and up until March 2024. Five different search engines were used (Medline (OVID), Scopus, Web of Science, Cochrane library and CINAHL). The search was expanded to include additional psychiatric conditions (e.g., suicidal ideation) and somatic conditions (e.g., tinnitus and chronic pain).

**Results:**

Of the 6509 identified articles, 39 meta-analyses met the inclusion criteria. In these meta-analyses 19 unique outcomes were represented. The most common outcome was symptoms of major depressive disorder, followed by symptoms of anxiety. Effect sizes for the comparisons against control conditions ranged between small (e.g., SMD = 0.10 for stress in employees) to large (e.g., SMD = 1.20 for depressive symptoms among older adults).

**Conclusions:**

ICBT can generally reduce symptoms of a wide range of conditions including both psychiatric and somatic conditions, as well as other mental health problems. This updated review of available meta-analyses also indicated that ICBT has been successful in treating symptoms in different subpopulations such as older adults and students. However, some knowledge gaps remain, including the use of ICBT for psychotic disorders, and the quality of the available meta-analyses’ points to a need for more stringent methodological procedures.

## Background

Since its emergence in the early 1970s, cognitive behaviour therapy (CBT) has become one of the most widely practiced and researched forms of psychotherapy worldwide.^1^ Due to the vast amount of research on CBT and how effective it has been shown to be in clinical trials, CBT is often regarded as a first-line choice in treatment recommendations and guidelines for many mental disorders.^2^ Meta-analyses support the notion that CBT is effective in treating a wide range of conditions, with average effect sizes in the moderate-to-large range compared to control groups both for anxiety disorders (effect size = 0.56),^3^ depression (effect size = 0.71),^4^ and other conditions such as insomnia (effect size = 0.98).^5^

One notable change in the delivery of CBT occurred in the 1990s, during which the first initiatives to computerize CBT were made.^6^ Computerized CBT subsequently evolved into what is now widely known as internet-delivered cognitive behaviour therapy (ICBT).^6^ ICBT is a form of CBT in which the content is presented online in the form of text, but also pictures, videos and interactive features.^7^ It differs from other kinds of internet-mediated psychotherapy, for example, therapy via video calls, in that the therapeutic elements (e.g., exercises and psychoeducation) are provided asynchronously and not by a therapist in real-time. ICBT can be delivered via devices with internet access, either through an app or a web platform. It can be delivered as pure self-help (where the client is provided access to the ICBT materials without any input from a therapist), with therapist support (where a therapist provides feedback and support on a weekly basis), or used in addition to face-to-face sessions with a therapist (blended ICBT). In comparison to previous delivery formats of CBT, such as individual therapy and group treatment, ICBT has several potential advantages. This includes increased flexibility for both patients and therapists in accessing and tailoring the treatment^8^ and increased anonymity for people who would not otherwise choose to access face-to-face services.^9^

Andersson and colleagues^10^ conducted the first narrative umbrella review of ICBT for adults with affective and anxiety disorders. After selecting and reviewing the most recent meta-analyses at the time of the search (in 2018), the authors summarized the effects of therapist-supported ICBT in treating five disorders: major depression, panic disorder, posttraumatic stress disorder (PTSD), social anxiety disorder and generalized anxiety disorder (GAD). Moderate-to-large effect sizes for treating these disorders were found, showing that therapist-supported ICBT is likely to be more effective than control conditions not involving active treatment ingredients. While encouraging, Andersson et al.^10^ reflected on a lack of meta-analytic reviews on specific phobias and obsessive-compulsive disorder (OCD). Further attention was also drawn toward evaluating the efficacy of transdiagnostic ICBT as well as the potential of ICBT to provide support for adults with more severe mental problems, such as schizophrenia.^10^ Since the publication of the previous umbrella review, many studies focused on applying ICBT more broadly have been published, both in terms of diagnostic groups and problem areas but also in specific subpopulations. This includes studies on the effectiveness of such interventions in clinical practice^11^ and the search for predictors of treatment success, such as demographic characteristics.^12^ Additionally, the need for guidance by a therapist is also a topic that has generated some attention, with unguided interventions potentially expanding the reach of ICBT even more, though questions have arisen about whether the two approaches are equally effective.^13^ Given the increasing number of studies and meta-analytic reviews in the field of ICBT,^14^ an updated umbrella review could provide updated insights into conditions and problems for which ICBT is effective, whether the effects are consistent across subgroups (e.g., do we see similar results for younger adults and older adults?) and whether factors such as guidance influence the outcome. These questions are highly relevant since COVID-19 had a notable effect on the use of digital health services,^15^ and with existing evidence of the pandemic's negative impact on mental health,^16^ has likely led to an increase in ICBT program development and research. Indeed, there is research to support this hypothesis, with some studies indicating a 140% increase in demand of ICBT for insomnia,^17^ and a 504% increased uptake for treatment of depression and anxiety disorders with ICBT.^18^ The data also indicated an increased adherence during this time, compared to pre-pandemic numbers, in the treatment of insomnia,^17^ but the opposite trend was seen in the treatment of depression and anxiety.^18^ Yet, it is not clear whether existing research gaps, as previously outlined by Andersson et al.,^10^ have been addressed. For example, whether ICBT research has focused more on severe mental health conditions. Another example is whether there are any other tendencies in the ICBT research field to address raised criticisms, such as the common use of waitlist control groups.^19^ The current study hoped to address these questions by providing an updated and expanded overview of the efficacy of ICBT for a broader range of adult psychiatric and somatic conditions.

## Methods

We conducted a systematic umbrella review of meta-analyses published since the publication of the prior umbrella review^10^ and expanded the search terms to also encompass somatic conditions, including hearing disorders and chronic pain, and some additional categories of psychiatric disorders, including eating disorders and schizophrenia spectrum disorders (for the somatic conditions the search was also conducted since the prior umbrella review, rather than since inception). Some terms and conditions were not included in the search, including substance use disorders. The preferred reporting items for systematic reviews and meta-analyses (PRISMA) checklist was used in conducting the review (see online Supplementary Materials).

## Search strategy and selection criteria

This umbrella review serves as an update on the prior review with its last search conducted in September 2018,^10^ which was registered at PROSPERO (Identifier: CRD42018106156) with information regarding the review question, the specified search strategy, the inclusion/exclusion criteria and the quality of evidence-assessment. The preregistration was revised to account for the updated search strategy and changes to inclusion/exclusion criteria. Two of the authors (A.K and I.B.) searched Medline (OVID), Scopus, Web of Science, Cochrane Library and CINAHL for systematic reviews and meta-analyses. The first search was conducted in May 2022 and two updated searches were conducted on 14 February 2023 and 1 March 2024. The search terms are detailed in appendix B of the Online Supplementary Materials. The inclusion and exclusion criteria and examples of study characteristics meeting/not meeting the criteria are listed in Table 1. As opposed to the previous narrative umbrella review, we chose to include all meta-analysis/systematic reviews that met the inclusion criteria. This meant that for some conditions (e.g., symptoms of major depressive disorder) there were more than one meta-analysis included. One of the authors (I.B.) conducted an initial search in 2022 and identified 217 records. Two of the authors (I.B. and A.K.) conducted the updated searches with 219 identified reports. For the latter search, the interrater reliability for inclusion in the review was κ = .91. Additionally, one of the authors (A.K.) screened the reports identified for inclusion during the initial search. Any disagreements were discussed, and if needed the senior researcher (G.A.) was consulted for a final decision.

**Table 1.** Inclusion/exclusion criteria.

Inclusion Criteria	Example of Meeting the Inclusion Criteria	Example of Not Meeting the Inclusion Criteria
Written in English	–	–
Published between 1 January, 2019 and 1 March 2024.	–	–
Contain an effect size estimate generated from pooling the quantitative results from randomized controlled trials (can be from either a meta-analysis or a systematic review that provide a pooled estimate of the effect sizes compared to an active or control condition).	A meta-analysis or systematic review that provide an overall estimate of treatment efficacy relative to either a control (e.g., a waitlist) or an active condition (e.g., compared to face-to-face CBT).	Systematic reviews with no pooled estimate available (e.g., that provide a forest plot of effects sizes for the interventions, but no overall estimate). Pooled estimates including results from uncontrolled pre-post designs.
Use the internet to provide CBT in an asynchronous manner via either a web platform or an app.	A meta-analysis only focusing on studies using a web-platform app to provide CBT for a specific condition.	Sole usage of a discussion forum or a web page with psychoeducation about a condition. Telepsychiatric solutions where regular face-to-face therapy is provided via phone calls or videoconference solutions. Computer games aimed at disseminating CBT principles.
Provide effect size estimates from some version of therapy-derived CBT.	Estimates from meta-analyses of internet-based CBT or third wave therapies (e.g., ACT)	Purely psychoeducational interventions with no broader theoretical basis (e.g., offering information about symptoms, but not any kind of information on how to handle them). Apps containing psychoeducation or techniques with no explicit theoretical basis (e.g., breathing exercises without a broader mindfulness-based cognitive therapy orientation). Estimates from studies on cognitive training.
Focused on mental health problems, either as the primary problem or as related to somatic illness.	Meta-analyses of internet-interventions targeting psychiatric symptoms or psychological distress stemming from somatic illness (e.g., cancer-related psychological distress).	Meta-analyses of internet-based intervention effects on medicine adherence.

Exclusion Criteria	Example of Meeting the Exclusion Criteria	Example of Not Meeting the Exclusion Criteria
Were purely bibliotherapeutic	Meta-analytic estimates based on studies of pure bibliotherapies with no internet component.	Meta-analytical estimates based on studies where part of the intervention was based on a bibliotherapeutic approach, but where part of the materials/communications are internet-based (e.g., older internet-based studies where participants printed out worksheets, but where communication with the therapist was conducted via emails or other asynchronous internet-based means).
Were based on blended forms of therapy where the face-to-face component made up more than 50% of the intervention materials/sessions.	Meta-analytic estimates based on studies where an internet-based intervention was used as an add-on to regular face-to-face therapy.	Meta-analytic estimates based on studies where an internet intervention served as the primary means of treatment, but occasional face-to-face sessions were used (e.g., four hour-long sessions during a ten-week intervention). Also, regular phone sessions that extends beyond the support function that the therapist has in guided therapies.
Did not provide separate estimates for interventions meeting the inclusion criteria and those that did not.	Meta-analyses which only provided a single estimate when some of the studies in the analyses did not meet all inclusion criteria (e.g., were conducted purely via a videoconferencing solution).	Meta-analyses which provided a separate estimate of the kind of intervention outlined in the inclusion criteria using a sensitivity analysis.

*Note*. CBT: cognitive behaviour therapy; ACT: acceptance and commitment therapy.

## Data extraction

Data extraction was performed by two of the authors (A.K. and I.B.) with A.K. extracting data from all the articles and I.B. extracting data from a subset of 14 articles. The interrater reliability was excellent, κ = .98, and the few discrepancies were checked and settled between the authors. For each article, we extracted the condition and target group, the number of comparisons used for the effect size estimate (the standardized mean difference, the odds ratio, or the unstandardized mean effect difference for fixed effects models), the total number of participants in the comparisons, the effect size for the treatment versus control comparison (the standardized mean difference; SMD), the 95% confidence interval for the comparison, the range of effect sizes for individual studies included in the meta-analysis and the heterogeneity estimate (*I*^2^; with confidence intervals when available). The *I*^2^ estimate was interpreted according to the rule-of-thumb of 0%–20% = no significant heterogeneity, 20%–40% = low heterogeneity, 40%–60% = moderate heterogeneity and >60% = high heterogeneity (20). For the effect size estimates, priority was given to the overall estimate. Where available we also extracted separate estimates for unguided ICBT versus control groups and guided ICBT versus control groups, including active comparisons between the two. With regard to the target condition, we used the primary outcome/outcomes as it were specified in the meta-analysis. In reviews where the authors specified multiple primary outcomes, we extracted all outcomes that were symptoms of psychiatric and/or somatic conditions. For consistency across all outcome measures, we extracted all effect size estimates in such a way that higher estimates favour ICBT over the control condition.

## Confidence of evidence

The confidence of evidence for each meta-analysis was assessed using the AMSTAR-2 rating system.^20^ To optimize the assessment of the reviews, we conducted a two-step evaluation. First, we assessed reviews using the AMSTAR-2 algorithm implemented on the instrument's website (https://amstar.ca/Amstar_Checklist.php). The algorithm generates a global score, ranging from critically low to high. This score is based on all 16 ratings and provides an overall estimate. Second, we devised a way of rating confidence based on a subset of the instrument domains that was considered to be relevant and important for psychological treatments more specifically. This rating was based on six of the items: (1) Establishment of methods prior to conduction of the literature search (i.e., preregistration), (2) How comprehensive the literature search was, (3) Adequate assessment of Risk of Bias (RoB), (4) Use of appropriate methods for pooling data and investigating heterogeneity, (5) Accounting for RoB when interpreting the findings and (6) An investigation of publication bias. To supplement these critical domains, we also used the ratings on the following noncritical domains: (1) Clear criteria set according to the PICO framework, (2) Study selection done in duplicate, (3) Data extraction done in duplicate, (4) Adequate description of studies, (5) Assessing the impact of RoB on the quantitative results, (6) Provide a discussion of the heterogeneity and (7) Reporting of conflicts of interest. Across the 13 items, we provided a rating of yes, partial yes, or no. For a rating of high or moderate, studies had to meet the criteria for all six critical domains. The difference between high or moderate was based on whether the article had a noncritical weakness in the other domains or not. No noncritical weaknesses meant a high confidence rating, and one or more eligible noncritical weaknesses led to a moderate confidence rating. One critical weakness led to a low rating, and more than one critical weakness led to a critically low rating. The authors A.K. and I.B. both assessed a subset of articles (*n *= 10). The interrater reliability across all items was good, κ = .80, and any discrepancies were discussed among all the authors. The author A.K. then assessed the remaining papers.

## Results

### Results of the literature search

**Figure 1.**
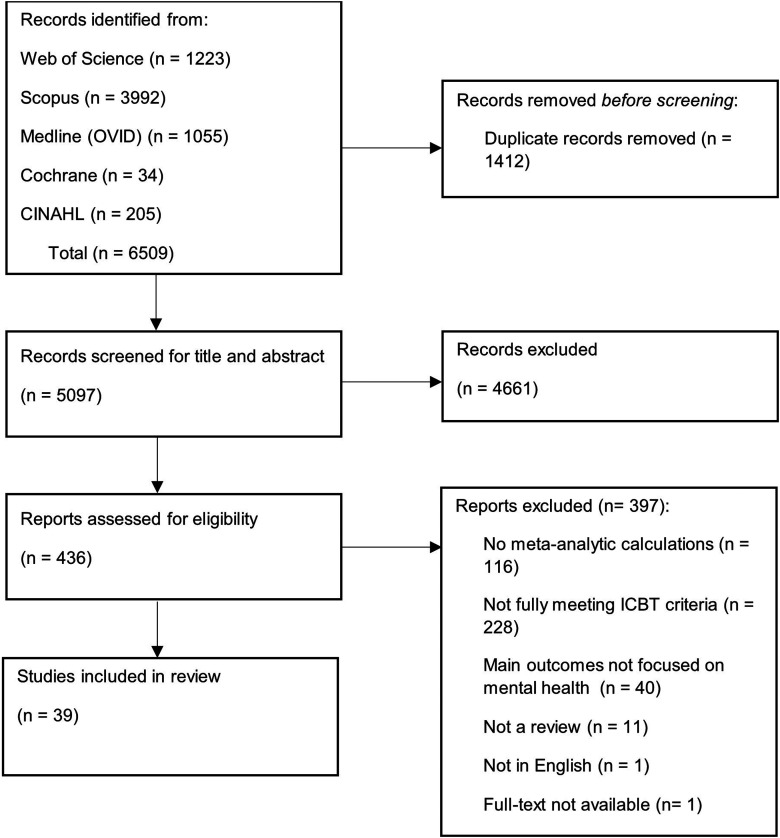
PRISMA Flow Diagram.

A flowchart of the search process is given in Figure 1. In total, 6509 articles were identified, out of which 436 were screened as full text. A total of 39 articles were included in the umbrella review.

### Characteristics of included studies

The study characteristics are listed in Tables 2–4. Full AMSTAR-2 ratings are available as part of the online Supplementary Materials.

**Table 2.** Recent meta-analyses of ICBT interventions targeting depressive symptoms, parasuicidal outcomes and grief.

Meta-analysis	Condition(s)	Notes	Comparisons Included	Total participants (*N*)	Effect Size Estimate [95% CI]	Effect Size Range	*I*^2^ [95% CI]	AMSTAR Rating Based on Critical Domains
Harrer et al. (2019)	Depressive symptoms in university students		19	10 583^a^	0.28 [0.15, 0.40]	−0.07 to 0.74	45% [6, 68]	Low
Loughnan et al. (2019)	Perinatal depressive symptoms		5	595^a^	0.60 [0.43, 0.78]	0.48–1.18;	0%	Critically low
Mehta et al. (2019)	Depressive symptoms in populations with chronic health conditions		25	3450	0.31 [0.27, 0.35]; 0.64 [0.49, 0.79] for guided treatments; 0.45 [0.27, 0.63] for unguided treatments	Not reported	81% for overall estimate	Low
Noh et al. (2023)	Depressive symptoms in adolescents and young adults		11	1704	0.35 [0.11, 0.60]	−0.19 to 1.25	82%	Critically low
de Oliveira et al. (2023)	Depressive symptoms in older adults		4	219	1.20 [0.81–1.60]	0.82–1.38	0%	Critically low
Roman et al. (2020)	Postpartum depression		6	635	0.54 [0.42, 0.72]	0.19–1.08	25%	Critically low
Ma et al. (2021)	Depressive symptoms in college students		12	800	0.42 [0.27, 0.57]	−0.42 to 1.79	7%	Low
Schiller et al. (2023)	Depressive symptoms	Remission and response rates only	6 for remission rate, 3 for response rate	619 for remission rate, 331 for response rate	OR for remission = 10.30 [5.70–18.60]; OR for response = 3.57 [0.86–14.78]	6.98–19.64 for remission; 1.18–11.69 for response	0% for remission rate, 79% for response rate	Low
Mu et al. (2021)	Postpartum depression		7	2277	0.64 [0.51, 0.81]; 0.69 [0.61, 0.79] for guided treatments; 0.37 [0.18, 0.76] for unguided treatments	0.26–0.91	60% for the overall estimate	Low
Thompson et al. (2021)	Depressive symptoms	Only ACT	27	4839^a^	0.38 [0.28, 0.49]	0.04–1.00;	35%	High
Klimczak et al. (2023)	Depressive symptoms	Transdiagnostic ACT treatments	27	10 730^a^	0.44 [0.32, 0.57]	−0.35 to 1.27	53%	Critically low
White et al. (2022)	Depressive symptoms in patients with chronic health conditions		39	6066^a^	0.34 [0.22–0.45]	−0.47 to 1.28	74% [65, 81]	High
Mancinelli et al. (2022)	Depressive symptoms in pregnant women		7	1633	0.36 [0.11, 0.61]	−0.11 to 0.99	65%	High
Gandy et al. (2022)	Depressive symptoms in patients with chronic pain		33	5778^a^	0.43 [0.33, 0.54]; 0.48 [0.37, 0.59] for guided treatments; 0.32 [0.11, 0.53] for unguided treatments	0.05–1.26	64% [48, 75] for the overall estimate	High
Lee et al. (2023)	Depressive symptoms in patients with insomnia		21	10 465	0.42 [0.28, 0.56]; 0.43 [0.26, 0.61] for unguided treatments	−0.06 to 0.84	82% for the overall estimate	High
Liu et al. (2022)	Depressive symptoms in patients with cancer		13	2179	0.27 [0.09, 0.44]; 0.36 [0.23, 0.49] for guided treatments; 0.14 [0.03, 0.26] for unguided treatments	−0.46 to 1.20	72% for the overall estimate	Critically low
Chan et al. (2022)	Depressive symptoms		14	1671	0.49 [0.27, 0.71]	−0.22 to 1.59	74%	Low
Stratton et al. (2022)	Depressive symptoms in employees		23	4961^a^	0.11 [0.06, 0.17]	−0.21 to 0.66	50%	Low
Sander et al. (2023)	Depressive symptoms in participants seeking help for suicidal ideation	IPD	8	1980	0.30 [0.17–0.43]	–	–	Low
Han and Kim (2022)	Depressive symptoms	Only behavioural activation	31	4124	0.35 [0.27, 0.44];	−0.14 to 1.04	34%	Critically low
Reins et al. (2021)	Subthreshold depressive symptoms	IPD	7	2186	0.39 [0.25–0.53]	N/A	N/A	High
Wagner et al. (2020)	Grief after bereavement		7	1257	0.54 [0.30, 0.78]	0.13–0.92	60%	High
Büscher et al. (2020)	Suicidal ideation		6	1567	0.29 [0.19, 0.40]	0.02–0.38	0%	High
Büscher et al. (2022)	Suicidal ideation	IPD	9	2037	0.25 [0.17, 0.32]	N/A	N/A	High
Torok et al. (2020)	Suicidal ideation		6	1234	0.14 [−0.06 to 0.34]	0.09–0.34	0% [0, 0]	High

*Note*. ACT: acceptance and commitment therapy; IPD: individual patient data meta-analysis.

^a^ When an exact number for the specific subgroup analysis is unavailable, the number of studies and/or the total number of participants denote the entire sample included in the meta-analysis.

**Table 3.** Recent meta-analyses of ICBT interventions targeting anxiety disorders, PTSD, obsessive-compulsive disorder and eating disorders.

Meta-analysis	Condition(s)	Notes	Comparisons Included	Total Participants (*N*)	Effect Size Estimate [95% CI]	Effect Size Range	I^2^ [95% CI]	AMSTAR Rating Based on Critical Domains
Bayrampour et al. (2019)	Perinatal anxiety symptoms		4	218	0.41 [0.11, 0.71]	0.23–0.61	0%	Critically low
Loughnan et al. (2019)	Perinatal clinical anxiety symptoms		4	1128^a^	0.54 [0.24, 0.85]	0.29–0.78	0%	Critically low
Liu et al., (2022)	Anxiety symptoms in patients with cancer		12	2079	0.37 [0.12, 0.62]; 0.38 [0.25, 0.51] for guided treatments; 0.17 [0.05, 0.28] for unguided treatments	−0.01 to 2.35	86% for the overall estimate	Critically low
Mehta et al. (2019)	Anxiety symptoms in populations with chronic health conditions		25	3450	0.45 [0.36, 0.54]; 0.57 [0.45, 0.69] for guided treatments; 0.54 [0.46, 0.62] for unguided treatments	Not reported	69% for the overall estimate	Low
Mancinelli et al. (2022)	Anxiety symptoms in pregnant women		3	299	1.96 [1.21, 2.72]^b^	1.10–2.29^b^	0%	High
Thompson et al. (2021)	Anxiety symptoms	Only ACT	20	4839^a^	0.24 [0.16, 0.33]	0.01–0.62	0%	High
Klimczak et al. (2023)	Anxiety symptoms	Transdiagnostic ACT treatments	22	10 730^a^	0.30 [0.17, 0.43]	−0.30 to 0.98	43%	Critically low
Oey et al. (2023)	Anxiety symptoms	Guided vs. unguided ICBT	13	1414	0.16 [0.03, 0.28] in favour of guided treatments	−0.22 to 0.53	24%	Moderate
White et al. (2022)	Anxiety symptoms in patients with chronic health conditions		31	6066^a^	0.22 [0.13–0.32]	−0.33 to 1.12;	63% [45, 74]	High
de Oliviera et al. (2023)	Anxiety symptoms in older adults		4	219	1.14 [0.72, 1.56]	0.72–1.96	1%	Critically low
Gandy et al. (2022)	Anxiety symptoms in patients with chronic pain		25	5778^a^	0.32 [0.24, 0.40]; 0.39 [0.31, 0.48] for guided treatments; 0.18 [0.07, 0.30] for unguided treatments	0.05–0.70	25% [0, 54]	High
Lee et al. (2023)	Anxiety symptoms in patients with insomnia		18	8928	0.29 [0.19, 0.40]; 0.29 [0.17, 0.41] for unguided treatments	−0.22 to 0.53	58% for the overall estimate	High
Sander et al. (2023)	Anxiety symptoms in participants seeking help for suicidal ideation	IPD	8	1980	0.16 [−0.04 to 0.36]	–	–	Low
Stratton et al. (2022)	Anxiety symptoms in employees		14	7949	0.11 [0.04, 0.19]	−0.15 to 0.76	10%	Low
Harrer et al. (2019)	Anxiety symptoms and eating disorder symptoms in university students		21 for anxiety outcomes, 9 for eating disorders	10 583^a^	Anxiety = 0.36 [0.23, 0.50]; Eating disorders = 0.52 [0.22, 0.82]	Anxiety = −0.55 to 0.96; Eating disorders = 0.16–0.68	Anxiety = 43% [5, 66]; Eating disorders = 61% [18, 81]	Low
Moghimi et al. (2021)	Binge eating disorder psychopathology		3	298	0.71 [0.22, 1.01]	0.38–1.17	77% [15, 64]	Critically low
Domhardt et al. (2020)	Panic disorder		16	1015	1.15 [0.74, 1.56];	0.25–2.61	79%	Low
Polak et al. (2021)	Panic disorder	Only active comparisons	10	744	0.02 [−0.16, 0.13]	−0.34 to 0.41	0%	Critically low
Cervin and Lundgren (2021)	Paediatric anxiety disorders		9	711	OR of remission for primary anxiety disorder = 4.73 [3.11, 7.29]; Youth-reported anxiety = 0.13 [−0.03, 0.28]; Caregiver-reported anxiety = 0.27 [0.04, 0.51]	Not reported	0% [0, 56] for remission data; 0% [0, 55] for Youth-reported anxiety; 41% [0, 88] for Caregiver-reported anxiety	Low
Guo et al. (2021)	Social anxiety disorder		20	1743	0.55 [0.55, 0.74]; 0.81 [0.63, 0.99] for guided treatments with experienced therapists; 0.82 [0.67, 0.97] for guided treatments with inexperienced therapists; 0.68 [0.41, 0.94] for unguided treatments	−0.40 to 1.28	72% for the overall estimate	Low
Lewis et al. (2019)	PTSD		8	560	0.60 [0.24, 0.97]	−0.05 to 1.82	76%	Low
Simon et al. (2021)	PTSD		10	608	0.61 [0.29, 0.93]; 0.78 [0.47, 1.09] for guided treatments; 0.09 [−0.22, 0.39] for unguided treatments	−0.05 to 1.82	69% for the overall estimate	High
O’Kearney et al. (2019)	ICBT versus face-to-face treatments for anxiety disorders	Only active comparisons	4 for social anxiety, 4 for panic disorder, 1 for specific phobia	885	0.01 [−0.32, 0.35] for social anxiety; −0.14 [−0.33, 0.05] for panic disorder; −0.22 [−0.91, 0.48] for specific phobias	−0.30 to 0.38 for social anxiety; −0.30 to 0.02 for panic disorder; N/A for specific phobia	64% for social anxiety; 0% for panic disorder; N/A for specific phobia	Critically low

*Note*. ACT: acceptance and commitment therapy; ICBT: internet-based cognitive behaviour therapy; IPD: individual patient data meta-analysis; PTSD: posttraumatic stress disorder.

^a^ When an exact number for the specific subgroup analysis is unavailable, the number of studies and/or the total number of number of participants denote the entire sample included in the meta-analysis.

^b^ The estimate is the unstandardized mean difference from a fixed effects model.

**Table 4.** Recent meta-analyses of ICBT interventions targeting symptoms of somatic conditions, sleep disorders and stress.

Meta-analysis	Condition(s)	Notes	Comparisons Included	Total Participants N	Effect Size Estimate [95% CI]	Effect Size Range	*I*^2^ [95% CI]	AMSTAR Rating Based on Critical Domains
Beukes et al. (2019)	Hearing impairment and tinnitus		15	1811	Hearing disability = 0.35 [−0.02, 0.72; tinnitus = 0.50 [0.37, 0.63]	Hearing disability = −0.37 to 0.93; Tinnitus = 0.04, 0.83	Hearing disability = 11%; Tinnitus = 0%	Low
White et al. (2022)	Distress in patients with chronic health conditions		15	6066^a^	0.49 [0.30–0.69]	Not reported	76% [62, 85]	High
Gandy et al. (2022)	Chronic pain disability in patients with chronic pain		40	5778^a^	0.28 [0.21, 0.35]; 0.38 [0.28, 0.47] for guided treatments; 0.16 [0.08, 0.23] for unguided treatments	0.00–1.25	33% [2, 55] for overall estimate	High
Lee et al. (2023)	Insomnia		19	8387	0.81 [0.65, 0.97]; 0.43 [0.26, 0.61] for unguided treatments	−0.26 to 1.34	80% for the overall estimate	High
Tsai et al. (2022)	Insomnia in young people		4	3970	0.58 [0.13–1.03]	−0.35 to 1.31	84%	High
Simon et al. (2023)	Insomnia	Network meta-analysis	16 for unguided ICBT, 10 for guided ICBT	12 544^a^	0.71 [0.24, 1.18] for guided treatments; 0.78 [0.38, 1.18] for unguided treatments	Not reported	Not reported	High
Mancinelli et al. (2022)	Sleep quality in pregnant women		3	289	0.40 [−0.08, 0.88]	−0.11 to 0.99	65%	High
Harrer et al. (2019)	Stress in university students		9	10 583^a^	0.20 [0.02, 0.38]	−0.35 to 1.26	78% [58, 88]	Low
Stratton et al. (2022)	Stress in employees		19	7949^a^	0.10 [0.04, 0.16]	−0.17 to 0.61	62%	Low
Svärdman et al. (2022)	Elevated stress		14	1831	0.78 [0.66, 0.90]	0.46–1.25	35% [32, 68]	Low

*Note*. ICBT: internet-based cognitive behaviour therapy.

^a^ When an exact number for the specific subgroup analysis is unavailable, the number of studies and/or the total number of number of participants denote the entire sample included in the meta-analysis.

### Depressive symptoms

The most common outcome in the meta-analyses published during the time frame was symptoms of depression. In total, 21 meta-analyses^21–41^ included symptoms of depression as the primary outcome and the effect sizes in these studies ranged from very small (SMD = 0.11)^34^ to large (SMD = 1.20).^23^ One meta-analysis^40^ analysed remission status and response status and found that ICBT led to a larger proportion of clients reaching remission as compared to control conditions but not to a significantly increased chance of treatment response. The populations covered in the studies included individuals with subclinical symptom levels,^31^ those with somatic disorders,^22,35^ or specifically targeted university students.^24,27^ Six of the meta-analyses on depressive symptoms (29%) as a primary outcome had a high AMSTAR-2 rating based on the critical domains. The rest were assessed as either low (38%, *n *= 8) or critically low (33%, *n *= 7).

### Suicidal ideation

Three meta-analyses focused on suicidal ideation as the primary outcome,^42–44^ and two out of the three showed a small but statistically significant reduction of suicidal ideation (range of SMDs = 0.25–0.29). The AMSTAR-2 ratings of the critical domains were high for all reviews.

### Grief

One meta-analysis focused on grief following bereavement.^45^ The estimate based on the seven comparisons of ICBT for this problem pointed to a moderate symptom reduction compared to the control conditions (SMD = 0.54). The meta-analysis had a high rating for the critical AMSTAR-2 domains.

### Anxiety symptoms

A total of 15 reviews used anxiety symptoms as the primary outcome.^22,24–29,33–37,39,46^ The effect sizes ranged from very small (SMD = 0.11)^33^ to large (SMD = 1.14).^35^ As with the reviews focusing on depressive symptoms, meta-analyses using anxiety symptoms as the outcome were conducted on different populations, including workplace-related settings,^33^ during pregnancy,^26,28,40^ and among persons with chronic health conditions.^22,29^ A third (33%) of the reviews (*n *= 5) had a high AMSTAR-2 rating of the critical domains. For the remaining reviews, one (7%) was rated as moderate, 27% (*n *= 4) were rated as low and 33% (*n *= 5) were rated as critically low.

### Anxiety disorders

Seven meta-analyses focused specifically on symptoms and/or diagnosis of an anxiety disorder or PTSD.^47–53^ Of these, two focused on panic disorder with or without agoraphobia, two focused on PTSD and one focused on social anxiety disorder. Additionally, the meta-analysis by Cervin and Lundgren^47^ used remission of the primary diagnosis as one of the primary outcomes and the review by O’Kearney et al.^51^ investigated the efficacy of ICBT relative to an equivalent face-to-face treatment for symptoms of social anxiety disorder, panic disorder and specific phobias. The disorder-specific anxiety outcomes showed significant reductions relative to passive control groups, with estimates ranging from moderate (SMD = 0.55)^49^ to large (SMD = 1.15).^48^ Compared to active control groups and equivalent face-to-face treatments, there were no statistically significant differences on any of the outcomes. For confidence rating according to the critical domains of the AMSTAR-2 system, 29% (*n *= 5) of the meta-analyses assessing symptoms of anxiety disorders and PTSD were graded as high. The majority were assessed as either low (41%, *n *= 7) or critically low (35%, *n *= 6).

### Stress

Three meta-analyses reported on stress-related outcomes, each in different settings.^24,33,54^ Effect sizes were all statistically significant and ranged from very small to small in a workplace setting and in university students (SMD = 0.10 and 0.20), to moderate-to-large in studies on samples with elevated stress levels (SMD = 0.78). All these reviews received a low AMSTAR-2 rating for the critical domains.

### Eating disorders

Two meta-analyses with a total of 12 comparisons assessed the effects of ICBT in reducing eating disorder psychopathology.^24,55^ One of these focused on eating disorders symptoms more broadly, and the other focused on symptoms of binge eating disorder specifically. The effect size for the comparison against the control group was moderate (SMD = 0.52) in a review focusing on university students, and moderate-to-large (SMD = 0.71) in a meta-analysis conducted on studies with community samples. The AMSTAR-2 ratings of the critical domains were low or critically low, respectively.

### Somatic disorders

With regard to somatic symptoms and disorders, we found one meta-analysis focused on symptoms of hearing impairment and tinnitus distress.^56^ This review reported a small and a moderate reduction (SMD = 0.35 for hearing disability, SMD = 0.50 for tinnitus distress), respectively, compared to control conditions. The AMSTAR-2 rating of the critical domains for the review was low. One meta-analysis had distress among patients with different chronic health conditions as a primary outcome and found a moderate effect size (SMD = 0.49) across the included comparisons.^29^ The AMSTAR-2 rating for the critical domains was high. Lastly, the search located one meta-analysis with pain disability as the primary outcome.^22^ The reported effect size was small (SMD = 0.28) and the AMSTAR-2 rating of critical domains was high.

### Sleep

For sleep disorders, three meta-analyses focused on insomnia symptoms^37,57,58^ and one on sleep quality in pregnant women.^28^ Significant reductions of insomnia symptoms were reported in all three meta-analyses, with moderate effect sizes (SMD = 0.58 and 0.71) in two^50,51^ and large effects (SMD = 0.81) in one.^38^ Comparable estimates were found for unguided and guided versions of ICBT. The meta-analysis reporting on the effects of sleep quality in pregnant women did not report a significant reduction relative to the control conditions.^29^ The AMSTAR rating for the critical domains were high for all four reviews.

### Guided and unguided versions of ICBT

Separate estimates for guided and unguided versions of ICBT can been seen in Tables 2, 3 and 4. A total of seven studies reported separate estimates for guided and unguided versions of ICBT in their studies.^22,25,29,30,49,52,57^ Additionally, one study reported estimates for unguided trials of ICBT but did not calculate an estimate for guided versions.^37^ Finally, one study reported on direct comparisons between guided and unguided ICBT targeting anxiety symptoms.^59^ Overall, all the estimates of guided ICBT suggested a significant effect of this treatment format on all kinds of symptoms (i.e., the confidence intervals did not contain zero). Four of the studies reported on depressive symptoms,^22,25,29,30^ three on anxiety symptoms,^22,25,29^ one specifically on symptoms of social anxiety disorder,^49^ one on PTSD,^52^ one on insomnia,^57^ and one on chronic pain.^22^ For unguided ICBT, significant symptom reduction was seen for depressive symptoms,^22,25,29,30,37^ anxiety symptoms,^22,25,29,37^ for social anxiety disorder,^49^ for insomnia,^57^ but not for PTSD.^52^ For direct comparisons, one study^59^ reported a small but significant difference (SMD = 0.16) between in favour of guided treatments targeting anxiety symptoms.

## Discussion

This umbrella review was an updated and expanded view of the evidence of ICBT for psychiatric and somatic conditions. In contrast to the previous umbrella review,^10^ this updated review included all meta-analyses for the selected conditions published between 2019 and March 2024 instead of focusing on the most recent reviews. The literature search resulted in over 6000 hits, out of which we included 39 unique meta-analyses. As a starting point, the results illustrate a continuous effort in developing and evaluating ICBT for a diverse set of populations, including those suffering from specific psychiatric symptoms such as suicidal ideation, but also from different demographic groups, such as students or older adults. Our findings also show that ICBT has been tested for several somatic conditions such tinnitus and other hearing problems, chronic pain and insomnia. In addition, as evident by the small-to-moderate effect sizes, ICBT can be regarded as a potential way to treat adults with psychiatric and somatic conditions. The expanded scope of these findings compared to the previous umbrella review also provides an indication the ICBT could be a valuable asset in regular care settings for other conditions than psychiatric disorders, such as chronic pain and work-related stress.

The effect sizes for anxiety disorders found in our review are generally similar to the ones found for more traditional forms of CBT,^3^ which is in line with the finding that ICBT and face-to-face treatments produce equivalent efficacy.^60^ For depressive symptoms, the estimates in many populations are somewhat smaller than the overall estimate for CBT^4^ although studies with both higher and lower effect sizes were found for the search. Importantly, when compared directly in the aforementioned study,^60^ the two modes of administering CBT did not differ significantly, which suggests clinical utility for this kind of treatment whether administered in person or via the internet. In relation to the previous umbrella review, ICBT continues to be used as a way of providing psychological treatments for a wide range of conditions. This is seen not only in the number of meta-analyses included in the review (which would simply be a consequence of including all studies, rather than selecting one or a few per condition) but also in the different target populations for whom ICBT has been applied. For example, a total of 21 meta-analyses used symptoms of major depressive disorder as a primary outcome and many of these targeted a distinct group suffering from these symptoms, such as patients with chronic health conditions or women with postpartum depression.

### The effects of ICBT

The inclusion of all meta-analytic reviews during the search period provides a broad overview of the effects of ICBT. The effect size estimates for the meta-analyses identified in our search commonly showed small or moderate effects across the different target groups and conditions, with all but two meta-analyses showing a significant treatment effect. Of note is that the effect sizes included in this umbrella review were generally somewhat smaller than the ones found in the previous review.^10^ Some methodological factors may have contributed to this finding. First, the estimates that were extracted in our review could include both passive and active control conditions, as well as guided and unguided versions of ICBT. The availability of therapist guidance has been linked to better treatment outcomes,^13^ which may explain part of the trend toward lower estimates in this updated review. Second, the choice to include all studies could also have led to the inclusion of studies in which participants may not have been recruited on the basis of having clinical symptoms levels, such as ICBT for mental health outcomes in a workplace setting.^33^ This could provide a ceiling effect on how much benefit can be derived from the intervention and subsequently influence the overall estimate presented in the meta-analysis. Though the diversity in populations provide important insights into the effects of ICBT in different settings and groups, this should be considered when interpreting the extracted data, particularly for the most common conditions (e.g., symptoms of major depressive disorder and anxiety symptoms). Another thing of note in relation to the effects is that a substantial number of the meta-analysis had a critically low rating of confidence according to the AMSTAR ratings. This is an important finding that points to a need for more stringent methodological procedures when conducting such analyses of ICBT interventions and its effects on different populations. These AMSTAR ratings should be kept in mind when interpreting the findings from the review. Overall, however, the small-to-moderate effect sizes suggest that ICBT can be effective in reducing symptoms of common mental health problems including depression, stress, and anxiety disorders such as panic disorder.

The need for therapist-guidance and whether it leads to better outcomes is a topic of discussion within the field of ICBT. Direct comparisons between guided and unguided treatments have been suggested to give an edge to treatments where a therapist provides support, both in terms of efficacy and for reducing the risk for dropout.^13^ The direct comparison included in our umbrella review also suggested that guided interventions had significantly steeper reductions of symptoms of anxiety^59^ although the effect size for the comparison was small. In general, unguided treatments were also shown to have significant effects for many different disorders and symptoms, including insomnia,^57^ social anxiety,^49^ and depressive symptoms.^22,29^ The exception to this trend was PTSD,^52^ where the unguided versions of ICBT did not provide the kind of benefits seen for guided treatments. To add to this, guided treatments generally had higher effect size estimates (although the opposite finding can be seen in insomnia) than unguided treatments. As these comparisons were not tested directly, it is not possible to draw definitive conclusions about the efficacy relative to one another, but the general findings could be taken as a sign that both modes of support are generally able to achieve a positive outcome when treating a broad number of conditions and problems. Future research should compare the two approaches directly in well-powered trials targeting different disorders and conditions to investigate whether the benefits of guidance are more pronounced in certain populations.

### The expanded use of ICBT

The expanded number of conditions we included resulted in estimates for eight conditions not found in the previous umbrella review: hearing impairment, tinnitus, chronic pain disability, insomnia, emotional distress, stress, eating disorder symptoms and sleep problems. Still, some of the proposed targets named in the previous umbrella review were also not found in this updated search which included search terms specifically for these disorders. This includes ICBT for obsessive-compulsive disorder and related disorders, and other psychiatric conditions such as bipolar disorder and schizophrenia. For the former, there are previous indications that ICBT is both effective and feasible,^61^ but an updated review of the field would be a welcome addition. For schizophrenia, the lack of meta-analyses most likely reflects a lack of studies conducted, although there are indications that ICBT can be feasible as an add-on to other psychological and pharmaceutical treatments.^62^ Additionally, we only found one review^37^ focusing on either transdiagnostic or tailored versions of ICBT like the meta-analysis found in the previous umbrella review.^63^ All in all, the findings in this updated review suggests that the effects of ICBT extends beyond the outcomes that that were the focus of the previous study. However, some of the same knowledge gaps that were identified then remain to this day and additional syntheses of available ICBT trials are important in these areas.

### Assessing outcomes of ICBT

An interesting observation is that the studies included in the meta-analytic reviews included almost exclusively used questionnaire data as outcomes. Only one review by Cervin and Lundgren^47^ used a diagnostic criterion for evaluating the efficacy of the treatments. Additionally, one meta-analysis focused on remission and response status in ICBT treatments of depressive symptoms.^40^ Although a diagnostic evaluation is not always possible, such as in the case of subthreshold symptoms,^31^ the use of more stringent criteria (e.g., a blinded diagnostic assessment) for evaluating treatment effects would be informative. This could also be important for providing indications for which forms of ICBT should be implemented in regular care settings, as patients within these settings might be more likely to meet the criteria for a psychiatric diagnosis. The finding that diagnostic status was not reported in all but one study highlights the need for providing this information both when detailing the results from individual RCTs and also when aggregating the results in systematic reviews and in meta-analyses.

### Strengths and limitations

In this umbrella review, we screened over 400 research articles after more than 6000 research hits were returned during the research process across five commonly used research databases. The umbrella review addressed some of the limitations found in the previous one, but there are others that should be acknowledged. First, the choice to include more than one meta-analysis per condition and to include additional conditions contributed to heterogeneity that made it harder to draw overarching conclusions about the effects of ICBT. Because the included reviews targeted different populations and had different inclusion criteria, even estimates for the same conditions and similar outcomes can be hard to compare across reviews. It is important to note that the aim of our umbrella review was not to provide an overall weighted average estimate of the effects of ICBT, but rather to provide insights into a selection of conditions and populations for which ICBT has been used. Future studies could synthesize the quantitative findings in ways that provide a unified effect size estimation, but this was considered out of scope for the present umbrella review. Second, the assessment of critical domains for the AMSTAR is not the conventional use of these ratings, and this should be accounted for when interpreting the findings. However, we believe that the rating system based on critical domains better represents the strengths and flaws found within the context of psychological treatments. The complete ratings can also be accessed in the supplementary materials, making this information available for interested readers. Third, due to the large number of identified reviews, we had to limit the screening to peer-reviewed publications searchable in commonly used academic databases. Further research efforts are encouraged in screening the gray literature as well as other sources, including manually screening reference lists. Fourth, while the principles of CBT can be disseminated in several ways, we choose to only include the studies that met the inclusion criteria for how ICBT has traditionally been developed and disseminated (e.g., using a web platform with asynchronous therapist contact). This choice of criteria allows for a more homogeneous set of studies to be included, which facilitates comparisons between studies and across conditions. However, this also means that other approaches that could be as effective are not represented in our results, whether they are based on different therapeutic orientations or other principles of dissemination. Lastly, the search terms did not encompass all psychiatric disorders and somatic conditions for which ICBT has been used. Because of this limitation, the absence of meta-analyses on ICBT targeting a specific condition in this umbrella review (e.g., substance use disorders) does not mean that such reviews do not exist. The scope of the search terms should be remembered while interpreting the findings from the systematic database searches. Expanding the search terms in future studies would allow for even broader conclusions than what is available in this umbrella review.

## Conclusions and future directions

This updated overview of 39 meta-analyses is in line with the conclusions made in the previous umbrella review and suggests that ICBT is an effective way to treat several mental health problems. The current review also provides an indication that this is the case for some somatic disorders, for example, chronic pain and tinnitus. Additionally, the diverse populations for which ICBT has been used successfully suggests that the treatment effects are generalizable to many subgroups, including among older adults and in student populations. Despite this, there is still a lack of knowledge about the efficacy of ICBT for several psychiatric conditions and a lack of robust information about for whom ICBT works best, both of which should be addressed in future studies. Many meta-analyses were also found to have methodological flaws, which highlights the need for improved procedures when conducting similar reviews and analyses in the future.

## Supplemental Material

sj-docx-1-dhj-10.1177_20552076241287643 - Supplemental material for Internet-delivered cognitive behaviour therapy for affective disorders, anxiety disorders and somatic conditions: An updated systematic umbrella review Supplemental material, sj-docx-1-dhj-10.1177_20552076241287643 for Internet-delivered cognitive behaviour therapy for affective disorders, anxiety disorders and somatic conditions: An updated systematic umbrella review by Anton Käll, Ieva Biliunaite and Gerhard Andersson in DIGITAL HEALTH

## List of abbreviations

ACT: Acceptance and commitment therapy
AMSTAR: A MeaSurement tool to assess systematic reviews
CBT: Cognitive behaviour therapy
DBT: Dialectical behaviour therapy
GAD: Generalized anxiety disorder
ICBT: Internet-delivered cognitive behaviour therapy
IPD: Individual patient data meta-analysis
OCD: Obsessive-compulsive disorder
PRISMA: Preferred reporting items for systematic reviews and meta-analyses
PTSD: Posttraumatic stress disorder
RoB: Risk of bias
